# Double-blinded, randomized controlled trial comparing real versus placebo acupuncture to improve tolerance of diagnostic esophagogastroduodenoscopy without sedation: a study protocol

**DOI:** 10.1186/1745-6215-12-52

**Published:** 2011-02-23

**Authors:** P Knebel, K Schwan, T Bruckner, CM Seiler, K Plaschke, K Streitberger, A Schaible, C Bopp

**Affiliations:** 1 Department of General, Visceral and Transplantation Surgery, University of Heidelberg, Germany; 2 Department of Anaesthesiology, University of Heidelberg, Germany; 3 Institute of Medical Biometry and Informatics, University of Heidelberg, Germany; 4 Department of Anaesthesiology und Pain Therapy, Inselspital Bern, Switzerland

## Abstract

**Background:**

Sedation prior to performance of diagnostic esophagogastroduodenoscopy (EGDE) is widespread and increases patient comfort. But 98% of all serious adverse events during EGDEs are ascribed to sedation. The S3 guideline for sedation procedures in gastrointestinal endoscopy published in 2008 in Germany increases patient safety by standardization. These new regulations increase costs because of the need for more personnel and a prolonged discharge procedure after examinations with sedation. Many patients have difficulties to meet the discharge criteria regulated by the S3 guideline, e.g. the call for a second person to escort them home, to resign from driving and working for the rest of the day, resulting in a refusal of sedation. Therefore, we would like to examine if an acupuncture during elective, diagnostic EGDEs could increase the comfort of patients refusing systemic sedation.

**Methods/Design:**

A single-center, double blinded, placebo controlled superiority trial to compare the success rates of elective, diagnostic EGDEs with real and placebo acupuncture. All patients aged 18 years or older scheduled for elective, diagnostic EGDE who refuse a systemic sedation are eligible. 354 patients will be randomized. The primary endpoint is the rate of successful EGDEs with the randomized technique. Intervention: Real or placebo acupuncture before and during EGDE. Duration of study: Approximately 24 months.

**Discussion:**

Organisation/Responsibility The ACUPEND - Trial will be conducted in accordance with the protocol and in compliance with the moral, ethical, and scientific principles governing clinical research as set out in the Declaration of Helsinki (1989) and Good Clinical Practice (GCP). The Interdisciplinary Endoscopy Center (IEZ) of the University Hospital Heidelberg is responsible for design and conduct of the trial, including randomization and documentation of patients' data. Data management and statistical analysis will be performed by the independent Institute for Medical Biometry and Informatics (IMBI) and the Center of Clinical Trials (KSC) at the Department of General, Visceral and Transplantation Surgery, University of Heidelberg.

**Trial registration:**

The trial is registered at Germanctr.de (DRKS00000164) on December 10^th ^2009. The first patient was randomized on February 2^nd ^2010.

## Background

More than 10 million gastrointestinal (GI) endoscopic procedures are performed every year in the United States only[1,2]. The standard use of systemic sedation to facilitate the performance of esophagogastroduodenoscopy (EGDE) and to increase patient comfort has contributed to the widespread use and acceptance of this procedure. But the perceived benefits of improved patient comfort and satisfaction afforded by parenteral sedation must be measured against the increased risk of adverse cardiopulmonary events and higher attendant costs[3]. Complications that arise from EGDE are usually associated with the use of systemic sedation and the dose given. More than 98% of serious adverse events in upper GI endoscopy like hypotension, aspiration and respiratory depression are ascribed to systemic sedation[4].

Recently, the first S3-guideline for sedation in GI endoscopy in Germany was published to improve patient safety, which demands additional trained personnel and endoscopy equipment[5]. Therefore, it is estimated that sedation and related issues account for up to 40% of total endoscopy costs including overhead and indirect costs[6]. In detail, an additional specialized nurse or physician is required to perform and monitor systemic sedation. Furthermore, systemic sedation impedes the rapid discharge from the hospital, resulting in absence of work of patients concerned.

**Figure 1:**
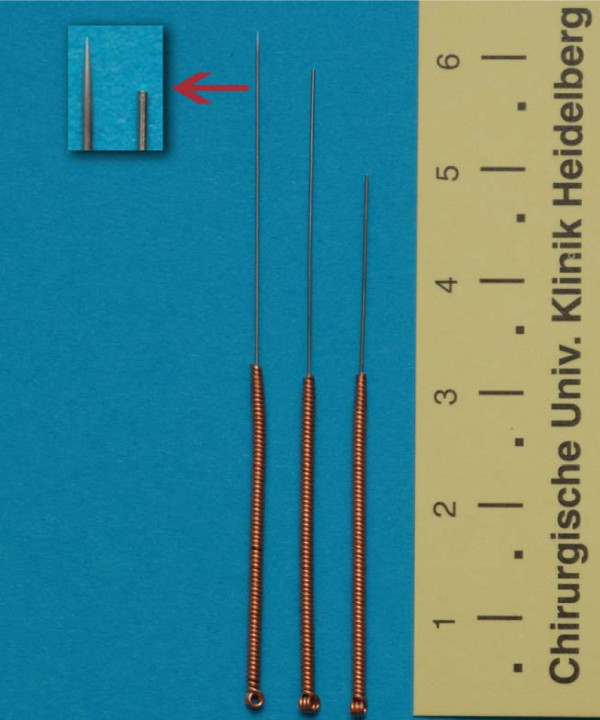
Image of real and placebo acupuncture needle system

Acupuncture has been used as a part of Traditional Chinese Medicine (TMC) for more than 2000 years[7]. Many studies have investigated the benefits and success of acupuncture in reducing pain for various acute and chronic diseases. However, most of them had methodological difficulties, e.g. the inclusion of an adequate control group[8]. A Cochrane review from 2009 on acupuncture for migraine prophylaxis reports that even acupuncture at the "wrong" place (sham-acupuncture) could have a significant effect on the primary endpoint[9]. Therefore, the use of a real placebo needle is necessary. With the introduction of a placebo acupuncture needle system some years ago a new and valid instrument to measure placebo effects became available (Figure 1)[10].

If the use of acupuncture could improve tolerance and examination quality of diagnostic EGDE without sedation it should be possible to increase the willingness of patients to undergo this examination without systemic sedation. Consequently, it would be possible to reduce the rate of serious adverse events due to systemic sedation and to lower the costs for personnel and material.

Up to now, only one double-blind, controlled trial with 90 patients undergoing EGDE was performed in 1978, using real versus sham-acupuncture (1 cm away from the acupuncture point) with 10 needles and electrical stimulation and showing that upper endoscopy was much easier and better tolerated after real acupuncture[11]. But this study misses a clearly defined primary endpoint and a detailed sample size calculation and has the disadvantage of using sham-acupuncture instead of a real placebo acupuncture technique in the control group. An adequately designed randomized double-blinded placebo controlled clinical trial with a well-defined primary endpoint and detailed sample size calculation comparing placebo acupuncture versus real acupuncture to improve success rates in EGDE has not yet been conducted.

## Methods/Design

### Aim of study

The objective of this trial is to compare the rate of successful EGDE (combination of patients' satisfaction and examination quality) in patients receiving pharyngeal anaethesia as well as either real or placebo acupuncture.

### Number of patients needed

The sample size calculation is based on the two-sided chi-square test for difference with respect to the primary endpoint.

**Figure 2:**
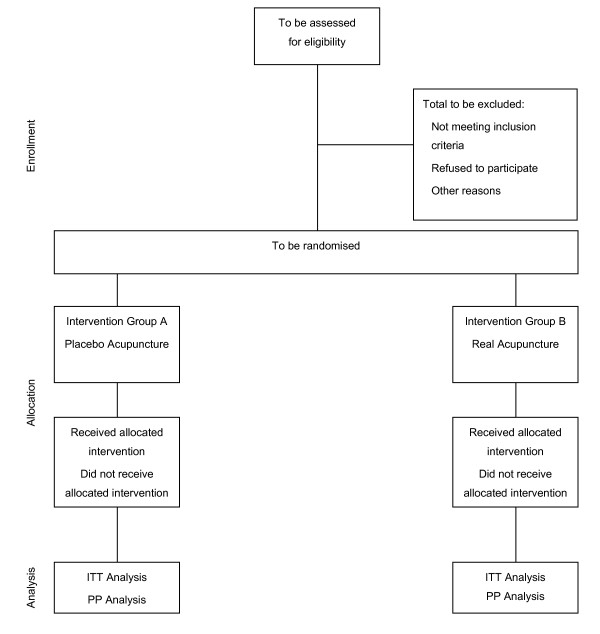
Flowchart according to CONSORT

A review of the previous literature identified a randomized controlled trial from Abraham et al. with a group of 419 patients comparing the rate of successful EDGE with and without sedation[12]. In both groups a pharyngeal anaesthesia was performed. This trial showed a successful examination rate of 46% in the group without sedation. A successful examination was defined as a composite score of patient satisfaction with the procedure as well as quality of the examination (technical adequacy) as assessed by the endoscopist. These were determined by the administration of standardized Likert scales (patient satisfaction on a scale of 1-5, quality of examination on a scale of 1-4). An EDGE examination was only counted as successful if the patient rated 1 or 2 on the satisfaction scale and the endoscopist rated 4 on the quality of examination scale. Based on the results from this large trial we decided to adapt the definition of successful EDGE for our trial and use the 46% success rate of the non sedated group as baseline for the sample size calculation. We believe that an increase of the success rate by 15% or more to > = 61% in the acupuncture group would be clinically relevant and therefore could impact clinical practice. To detect this difference at a type I error rate of α = 0.05 (two-sided) with power 1-β = 80%, a sample size of n = 173 evaluable patients per group is necessary (SAS 9.1 proc power). The drop out rate within the intervention is expected to be about 2% overall. Therefore, another 8 patients in total have to be randomized to obtain the required number of evaluable patients. The total number of patients needed to be randomized is therefore 354 (Figure 2).

### Eligibility

Inclusion criteria

• Age equal or greater than 18 years

• Patients scheduled for elective diagnostic EGDE without systemic sedation

Exclusion criteria

• Patients classified ASA V

• Participation in another clinical trial which could interfere with the primary endpoint of this study

• Expected lack of compliance

• Impaired mental state or language problems

• Patients with known allergy to lidocaine anaesthetic spray or acupuncture needle material

• Necessity of systemic sedation

• Emergency procedures

• Pregnancy

Subject withdrawal criteria

• At their own request or at request of the legal representative

• If, in the investigator's opinion (physician performing the acupuncture or physician performing the examination), continuation of the trial would be detrimental to the subject's well-being (e.g. strong pain at the insertion points, allergical reactions, other independent acute health problems).

All withdrawn patients will be reported in the final results to guarantee maximum transparency.

### Consent

The ACUPEND- Trial will be conducted in accordance with the protocol and in compliance with the moral, ethical, and scientific principles governing clinical research as set out in the Declaration of Helsinki 1989[13] and Good Clinical Practice http://www.ema.europa.eu/docs/en_GB/document_library/Scientific_guideline/2009/09/WC500002874.pdf. The protocol was approved by the Ethics Committee of the University of Heidelberg (S-273/2009). All patients scheduled for an elective diagnostic EGDE in the Interdisciplinary Center of Endoscopy (IEZ) of the Department of General, Visceral and Transplantation Surgery and the Department of Internal Medicine of the Universitiy of Heidelberg will be screened, informed about the ACUPEND trial and included by physicians of the IEZ supervising in this trial. The study procedure, risks, benefits and data management will be clarified in detail before the patients are asked to give their informed consent.

#### Randomization and procedures for minimizing bias

The study protocol was designed according to the Standards for reporting Interventions in Clinical Trials Acupuncture (STRICTA)[14].

##### Minimizing bias

To achieve comparable groups for known and unknown risk factors, randomization will be performed as a block randomization with alternating block size. Allocation to treatment group will be performed by sealed consecutively numbered envelopes prepared by the Institute of Medical Biometry and Informatics of the University of Heidelberg (IMBI). Randomization will be carried out after the patient has signed the informed consent form and will be documented in the case report file of every patient. The allocation will be controlled by a monitor.

##### Minimizing treatment bias

All physicians who participate in this trial will be trained and updated every 3 month to improve comparable treatment of patients.

Special manuals will be used in the Interdisciplinary Center for Endoscopy (IEZ) to reduce errors. Acupuncture will be performed by physicians of the department of the Interdisciplinary endoscopy center (IEZ) who were trained by experienced acupuncturists with level B diploma and who performed at least 20 acupuncture procedures explained in the intervention chapter under supervision. Furthermore the PS3 device will be used to support the localisation of the correct acupuncture points by measuring loss of skin resistance.

##### Minimizing measurement bias

A study nurse or physician will document and monitor the procedure in the endoscopy and recovery room. Before the endoscopic procedure is performed the acupuncturist will open the allocation envelope. The physician performing the EGDE and the assistant nurse will not be informed about the allocation. Once patients are randomized they will be included into the analysis according to the principle of intention to treat.

#### Study treatment

Patients who are scheduled for EGDE will be screened and informed by a member of the IEZ about the trial before their EGDE procedure. After signing the informed consent form the patients will be prepared by staff nurses for the procedure as usual. A study nurse or physician will be present to monitor and document the procedures (Table 1).

**Table 1:** Study Visit Schedule

	Day of screening and intervention
Past medical history*	X

Informed consent	X

Personal data**	X

Examination of primary endpoints:	X
• Success of EGDE	

Examination of secondary endpoints:	
• Heart rate	X
• Blood pressure	X
• Oxygen saturation	X
• Duration of examination	X
• Willingness to repeat examination under same conditions	X
• Periinterventional complications	X

Safety criteria AE, SAE (2.6)	X

*study-relevant past medical history, past surgical history

** height (cm), weight (kg), gender, smoking customs, chemotherapy, existence of false teeth

All patients will be positioned on a stretcher lying on their backs with 30 degrees reverse Trendelenburg's position. Standard monitoring with SaO_2 _and non-invasive blood pressure will be established. Pharyngeal anaesthesia will be performed with a topical xylocaine spray (AstraZeneca, Germany) in all patients by a nurse or physician.

##### According to the allocation the procedure will be continued

We based the acupuncture point selections on Traditional Chinese Medicine meridian theory to improve tolerance and examination quality of diagnostic EGDE[15].

##### Intervention-group A (placebo acupuncture)

**Figure 3:**
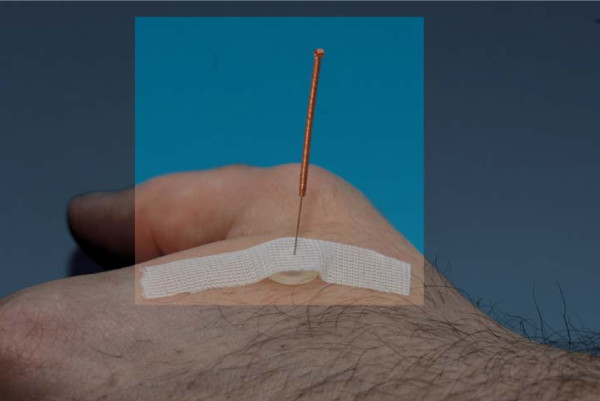
Inserted acupuncture needle

All patients will receive placebo acupuncture at the following points according to the procedure of the Streitberger placebo acupuncture needle system (Figures 1 and 3) [10]:

KG 24   Chengjiang   middle line (to reduce choking)[15]

Pericard 6   Neiguan   bilateral   (to reduce gastroenteral motility)[16]

Di 4   Yangxci   bilateral   (to reduce nausea and vomiting)[17]

Half to one inch 32G needles (0.32 × 30 mm stainless steel needle from asia med company, Munich, Germany) will be placed and left in their position for five minutes prior and throughout the endoscopic procedure.

##### Intervention-group B (real acupuncture)

After the described preparation patients will receive a real acupuncture at the following acupuncture points:

KG 24   Chengjiang   middle line   (to reduce choking)[15]

Pericard 6   Neiguan      bilateral      (to reduce gastroenteral motility) [16]

Di 4   Yangxci      bilateral      (to reduce nausea and vomiting)[17]

Half to one inch 32G needles (0.32 × 30 mm stainless steel needle from asia med company, Munich, Germany) will be placed into these points, stimulated for 1-2 seconds to induce de qi sensation and left in this position for five minutes prior and throughout the endoscopic procedure.

To perform EGDE all patients in both groups will be turned on their left side still with a 30 degree reverse Trendelenburg's position.

#### Primary and secondary endpoints

##### Primary endpoint

The primary endpoint will be the rate of successful diagnostic EGDE.

##### Definition of the primary endpoint

Rate of successful elective diagnostic EGDE is defined as a composite score of patient satisfaction with the procedure as well as quality of the examination (technical adequacy) as assessed by the examiner. These were determined by the administration of standardized Likert scales.

##### Assessment of the primary endpoint

Immediately after the elective diagnostic EGDE, the examiner scores the technical adequacy of the examination. Each anatomic area (esophagus, stomach, duodenum up to the second stage, and proximal stomach viewed in retroflexion) that was adequately viewed receives a score of 1 versus 0 if inadequately viewed, for a maximum score (4/4) if all four main anatomic areas of the examination were well visualized. The result will be documented in the CRF with tick boxes by the physician who performed the examination.

The patients are asked to rate their satisfaction with the examination after the completion of EGDE, prior to being told the results of their procedure and prior to discharge from the recovery room, on a Likert scale from 1 = acceptable to 5 = unacceptable. The results will be documented in the CRF with tick boxes by a nurse or physician. An EGDE will be counted as success if patients' satisfaction is 1 or 2 on the Likert scale and the examination quality score is 4/4[12,18-20].

##### Secondary endpoints

The willingness to repeat the procedure is defined as readiness of the patient to repeat the examination under the same conditions and will be recorded in the CRF with tick boxes (yes/no) by a nurse or physician. Heart rate (beats per minute), blood pressure (mmHg) and oxygen saturation (per cent) will be assessed before EGDE, after passage of the larynx and after removal of the endoscope. The results will be documented in the CRF by a nurse or physician. The duration of the examination in minutes from insertion to removal of the endoscope will be documented in the CRF. Furtheremore periinterventional complications as described and defined in Table 2 will be documented.

**Table 2:** Definitions of periinterventional complications:

Complication	Definition
Haematoma	Clinical diagnosis

Bleeding	Clinical diagnosis

Nerve irritation	Clinical diagnosis

Bradycardy	Heart rate < 60 beats per minute

Hypotension	Blood pressure < 90 mmHg systolic

Low oxygen saturation	Oxygen saturation < 92%

Aspiration	Clinical diagnosis and radiological findings

Wound infection	Clinical diagnosis

#### Safety aspects

##### Specification of safety variables

The correct placement of the needles will be checked and if necessary replaced by the physician who performed the acupuncture right before the examination. After endoscopic examination the needles will be checked again. If some of the needles are not in place after examination it will be documented in the CRF.

##### Concomitant medication

Every patient can be switched to systemic sedation at anytime to successfully complete the EGDE if necessary or at the patient's wish. The conversion will be documented in the CRF and the patient's satisfaction will be counted as 5/5 (unacceptable) on the Likert scale. Therefore, the need for systemic sedation is defined as unsuccessful EGDE for both intervention groups.

##### Past medical history

Prior and concomitant illness of the patients will be documented in the CRF.

##### Adverse events and serious adverse events

AEs will be reported to the principal investigator in regular intervals during the course of the study. SAEs will be documented on a special SAE form in the CRF and will be reported to the principal investigator within 24h.

#### Analysis

##### Analysis sets

Each patient's allocation to the different analysis populations (full analysis set (FAS) according to the intention-to-treat (ITT) principle, per protocol (PP) analysis set, safety analysis set) will be defined prior to the analysis. The allocation will be documented in the statistical analysis plan. During the data review, deviations from the protocol will be assessed as "minor" or "major". Major deviations from the protocol will lead to the exclusion of a patient from the PP analysis set.

##### Confirmatory analysis

The null hypothesis H is assessed by testing the intervention effect in a primary analysis using a two-sided chi-square test. In a secondary analysis a binary logistic regression model that takes into account the covariates "intervention" (placebo/acupuncture), age (< 65,≥ 65) and gender will be used. A two-sided type I error rate of α = 0.05 will be applied to the primary and secondary analysis.

Confirmatory analysis will be primarily based on the FAS which is consistent with the intention-to-treat (ITT) principle, by including all patients who were randomized into the two groups. This approach reflects the idea that the study should correspond to the conditions in clinical practice as closely as possible.

##### Further analysis

In addition to the evaluation of the FAS, a PP analysis will be performed including all randomized patients without major protocol violations.

The secondary variables will be analyzed in a descriptive manner by tabulation of the measures of the empirical distributions. According to the scale level of the variables, means, standard deviations, medians, 1^st ^and 3^rd ^quartiles as well as minimum and maximum or absolute and relative frequencies, respectively, will be reported. Descriptive p-values of the corresponding statistical tests comparing the treatment groups and associated 95% confidence intervals will be given.

##### Homogeneity of the treatment groups

The homogeneity of the treatment groups will be demonstrated descriptively using the demographic data and the baseline values. All statistical analyses will be performed using SAS^® ^software, Version 9.1 (or higher) of the SAS System for Unix (SAS Institute Inc., Cary, NC, USA).

#### Study organization

After approval of the protocol by the local ethics committee of the University of Heidelberg, the trial was internationally registered at Germanctr.de (DRKS00000164). All patients scheduled for elective, diagnostic EGDE in the Interdisciplinary Endoscopy Center (IEZ) who refuse sedation will be referred to and screened by members of IEZ or the Clinical Study Center Surgery (KSC). The result of the screening will be recorded in the screening-log.

Approximately 4300 patients per year undergo an EGDE including about 900 patients without systemic sedation in the Interdisciplinary Center for Endoscopy (IEZ) of the Universitiy of Heidelberg. The estimated time frame to randomize 354 patients is 20 months.

Sponsor of the ACUPEND trial is the University Hospital of Heidelberg.

The independent data management and statistical analysis will be carried out by the Institute of Medical Biometry and Informatics (IMBI) of the University of Heidelberg according to a prespecified Statistical Analysis Plan.

The principal investigator has the right to terminate the trial and to remove all trial material from the trial centre at any time in consultation with the Clinical Study Team Leader and the Biostatistician. Reasons that may require a termination of the trial include the following:

• The incidence or severity of adverse events in this trial indicates a potential health hazard caused by the study treatment

• It appears that patient's enrolment is unsatisfactory with respect to quality or quantity or data recording is severely inaccurate or incomplete

• External evidence that renders the necessity to terminate the trial

#### Financial support

The trial will be sponsored by the regular research budget (State of Baden-Württemberg) of the Clinical Study Center Surgery (KSC), Department of General, Visceral and Transplantation Surgery of the University of Heidelberg and the IEZ.

## Competing interests

The authors declare that they have no competing interests.

## Authors' contributions

PK, CB, CMS, KP and AS are responsible for the study design, definitions of the primary and secondary endpoints and preparation of the protocol. TB is responsible for the sample size calculation. KS carried out the literature research.
